# Evolutionary Insight into the Trypanosomatidae Using Alignment-Free Phylogenomics of the Kinetoplast

**DOI:** 10.3390/pathogens8030157

**Published:** 2019-09-18

**Authors:** Alexa Kaufer, Damien Stark, John Ellis

**Affiliations:** 1 School of Life Sciences, University of Technology Sydney, Ultimo, NSW 2007, Australia; john.ellis@uts.edu.au; 2 Department of Microbiology, St Vincent’s Hospital Sydney, Darlinghurst, NSW 2010, Australia; damien.stark@svha.org.au

**Keywords:** Trypanosomatidae, kinetoplast, second-generation sequencing, third-generation sequencing, alignment-free phylogenetics

## Abstract

Advancements in next-generation sequencing techniques have led to a substantial increase in the genomic information available for analyses in evolutionary biology. As such, this data requires the exponential growth in bioinformatic methods and expertise required to understand such vast quantities of genomic data. Alignment-free phylogenomics offer an alternative approach for large-scale analyses that may have the potential to address these challenges. The evolutionary relationships between various species within the trypanosomatid family, specifically members belonging to the genera *Leishmania* and *Trypanosoma* have been extensively studies over the last 30 years. However, there is a need for a more exhaustive analysis of the Trypanosomatidae, summarising the evolutionary patterns amongst the entire family of these important protists. The mitochondrial DNA of the trypanosomatids, better known as the kinetoplast, represents a valuable taxonomic marker given its unique presence across all kinetoplastid protozoans. The aim of this study was to validate the reliability and robustness of alignment-free approaches for phylogenomic analyses and its applicability to reconstruct the evolutionary relationships between the trypanosomatid family. In the present study, alignment-free analyses demonstrated the strength of these methods, particularly when dealing with large datasets compared to the traditional phylogenetic approaches. We present a maxicircle genome phylogeny of 46 species spanning the trypanosomatid family, demonstrating the superiority of the maxicircle for the analysis and taxonomic resolution of the Trypanosomatidae.

## 1. Introduction

Protozoan flagellates of the trypanosomatid family (syn. Trypanosomatidae) are obligate, unicellular parasites that infect a wide array of vertebrates, invertebrates and plants [1,2]. Protozoan parasites of this diverse family are predominately monoxenous (i.e., those restricted to a single, mainly invertebrate lifecycle), however the better known dixenous members (i.e., those with an invertebrate and vertebrate host) such as *Leishmania* and *Trypanosoma* are the causative agents of some of the most important neglected tropical diseases (NTD) including leishmaniasis, human African sleeping sickness (HAT) and Chagas disease [3,4,5]. The trypanosomatid family belongs to a distinct evolutionary lineage of eukaryotes within the class Kinetoplastida [6]. The current stance on kinetoplastid phylogeny is that the dixenous organisms evolved from the monoxenous members of the Trypanosomatidae several times throughout history, leading to the independent emergence of the genera *Trypanosoma*, *Phytomonas* and a group that unites *Leishmania*, *Porcisia* and *Endotrypanum* [5,7,8].

Traditionally, phylogenetic relationships within the kinetoplastids, including the trypanosomatid family have been predominately based on the analysis of the SSU rRNA genes [7,9]. The evolutionary rate of substitutions in genes is considered one of the most important factors that influence the informativeness and robustness of phylogenetic analyses [10]. Advancements in molecular biology have demonstrated that slow-evolving genes (like the SSU rRNA) are not reliable markers for the deep level resolution of species in order to determine the exact branching within the trypanosomatid family [7]. Nowadays, a multi-marker approach using concatenated sequences of multiple genes are becoming the preferred choice and are routinely employed for the phylogenetic inference of related organisms [5].

Exclusive to kinetoplastid protozoans, the mitochondrial DNA of trypanosomatids is an extensive network of DNA circles which are condensed into a periflagellar structure known as the kinetoplast (kDNA) [11,12]. The kDNA consists of approximately 10,000 minicircles ranging from 0.5 kb to 10 kb and 20–50 larger maxicircles ranging from 20 to 40 kb [13,14]. Recent analyses of *Trypanosoma brucei* demonstrated that the kDNA constitutes only 4.18 megabase pairs (Mbp) of the trypanosomatids’ total genome size of 77.7 Mbp [12]. Containing the mitochondrial homologues common to other eukaryotes, the maxicircle kDNA consists of two regions; a coding region containing the protein-coding genes and a highly repetitive non-coding region termed the divergent region (DR) [15,16,17]. In recent years, the maxicircle genome has become well-established as a superior taxonomic marker for the evolutionary analyses between related organisms of the Leishmaniinae and *Trypanosoma* [11,18,19]. Following suit with this rationale, the maxicircle kDNA should provide a more resolute model to investigate the genetic relationships between the entire trypanosomatid family, providing an all-encompassing analysis on the origins and biology of the Trypanosomatidae.

Over the past decade, second-generation sequencing (SGS) and third-generation sequencing (TGS) have become the leading technologies in the typing and evolutionary analyses of related organisms [20]. While short-read SGS techniques such as Illumina platforms have revolutionised biomedical research, their limitations, specifically their short-read (SR) lengths, make them inadequately suited for the assembly of complex and highly repetitive genomic regions [21]. Long-read TGS techniques such as PacBio offer longer read lengths (average >10 kb) than those of SGS, making it an ideal candidate for complex genomes [22]. However, a long-read (LR) length is hindered by a higher error rate of approximately 11–15% compared to that of SGS [22,23]. Despite this, the advantages of SGS and TGS are complementary, offering an alternative ‘hybrid’ strategy that makes use of both technologies to overcome the drawbacks of each method alone. These hybrid methodologies using long (and inaccurate) and short (and accurate) have proven to be extremely useful in producing high-quality, accurate assemblies [23,24].

Next-generation sequence data has paved the way for the use of phylogenomic approaches for the analysis of evolutionary relationships. Phylogenomics is the junction between evolution and genomics, using the comparative analysis of genome scale data for the reconstruction of evolutionary histories between organisms [25]. However, analyses with larger datasets across a wider breadth of taxa are becoming increasingly computationally infeasible [26]. The vast increase in genetic information necessitates the exponential growth in bioinformatic methods and expertise required to understand such immense quantities of genome-scale data. Thus, for large-scale analyses of genomes (i.e., phylogenomics), alignment-free (AF) methods of phylogenetic inference have been increasingly employed over the last few years [27]. Alignment-free software was first introduced nearly a decade ago but received little attention due to the traditional belief of its inferior resolution to multiple sequence alignment (MSA) based methods. However, due to the immense quantities of genome-scale data being produced, recent years have seen a surge in publications using AF applications for the phylogenetic analysis of organisms [20,26,27,28,29,30,31,32].

Traditionally, MSA-phylogenetics is based on the correspondence of individual nucleotides or amino acids that are in the same order between the species analysed [33]. Alignment-based methods for the most part yield excellent results when the dataset of sequences can be reliably aligned, however in certain instances, alignment-based sequence analyses can become problematic [34]. When sequences are divergent (i.e., analysis of the entire trypanosomatid family), the accuracy of sequence alignments decreases rapidly when the sequence identity falls below a certain critical point [33]. Second, alignment-based protocols are computationally intensive on a genome-scale [33], which is problematic with the increasing trend of whole-genome sequencing (WGS) replacing the use of single or few genes for the phylogenetic inference of related organisms.

The increasing trend of alignment-free sequence analyses offers an alternative approach for large-scale comparisons that may have the potential to address these limitations. One such alignment-free method introduced by Sims et al. [35] uses a measure based on the divergence between feature frequency profiles (FFPs), where the features (syn. word length) called k-mers are short nucleotide or amino-acid sequences of length *k*. For example, two sequences *x* = TTAAGG and *y* = AAGGCC and a feature length or k-mer size of three nucleotides produces *K*$\frac{x}{3}$ = (TTA, TAA, AAG, AGG) and *K*$\frac{y}{3}$ = (AAG, AGG, GGC, GCC) [33]. The frequency of these sub-sequences of a defined length or k-mers (*k*) are counted via a sliding frame implementation and used to calculate distance scores, which are subsequently used to generate a phylogenetic tree [35].

The aim of this paper is two-fold. First, the validation of the FFP protocol using the trypanosomatid family, in order to determine the limitations and variables in which the method provides reliable and robust results. Second, the application of this method to establish and summarise the current evolutionary relationships of the Trypanosomatidae. Rather than using data from the entire kinetoplast (minicircles and maxicircles), we focus on assembled genomes of the maxicircle only. In our previous study, we demonstrated that the maxicircle represents a superior, phylogenetically informative marker for studying the evolutionary relationships of the Leishmaniinae [19]. In this study, we expand on this concept and use the maxicircle kDNA to analyse not only the members of the Leishmaniinae, but representatives of the entire trypanosomatid family to provide an updated scheme for the taxonomic classification and resolution of the Trypanosomatidae.

In this study we applied a traditional MSA and AF method to the coding region of the maxicircle from a dataset of 46 trypanosomatid species. In addition to sequences already available from online databases and our previous study [19], twelve maxicircle genomes were assembled using both SR and SR/LR hybrid assemblies from raw Illumina and PacBio sequence data freely available from online databases (Sequence Read Archive). As a contribution to these efforts, this study contributes to our understanding of the phylogenomic relationships of the trypanosomatid family, demonstrating the power and robustness of the alignment-free analysis method based on the divergence between feature frequency profiles.

## 2. Materials and Methods

### 2.1. Samples

The trypanosomatid species used in this study are listed in Supplementary Materials S1 (S1 file).

### 2.2. Genome Assembly and Sequence Analysis

To obtain the complete maxicircle genome, processed reads were assembled from WGS data freely available through the Sequence Read Archive (SRA) on NCBI. Four paired-end Illumina and eight hybrid assemblies used a combination of long-read (PacBio) and short-read (Illumina) data to generate a hybrid contig using SPAdes version 3.12.0 [36]. For the hybrid assemblies, the datasets analysed were selected on the basis that both Illumina and PacBio sequence reads were available from the same author/provider and available for identical isolates/species. The maxicircle kDNA assembled from the Illumina assemblies (*Leishmania macropodum, Leishmania martiniquensis, Trypanosoma grayi* and *Phytomonas françai*) and hybrid assemblies (*Leishmania aethiopica, Leishmania amazonensis, Leishmania braziliensis, Leishmania guyanensis, Leishmania infantum, Leishmania mexicana, Leishmania tropica* and *Trypanosoma brucei rhodesiense*) were identified through BLAST analysis using NCBI BLAST software [37]. Annotation, gene identification and sequence analysis of the maxicircle from whole-genome sequencing was completed using Geneious version 11.0.2 [38]. The GC% content and GC skew was visualised with the software DNAPlotter [39].

In a given DNA sequence, we measure skewed mononucleotide frequencies by:

$$\mathrm{ATS}\;=\;\frac{f_{A\;-\;}f_{T}}{\;f_{A}\;+\;f_{T}}\;\mathrm{and}\;\mathrm{GCS}\;=\;\frac{f_{G\;-\;}f_{C}}{\;f_{G}\;+\;f_{C}}$$

where $f_{N\;}$ denotes the observed frequency of nucleotides A, T, G and C [40].

### 2.3. Comparative Analysis of the Non-Coding Divergent Region of the Trypanosomatid Family

Self-dot plot analyses were generated in Geneious using the EMBOSS 6.5.7 software Dottup suite add-on [38]. The repetitive portion of the non-coding divergent region of 42 trypanosomatid species were visualised as a dense block of identity in a dot matrix homology search comparing each species against its own DNA sequence. The divergent region of *Endotrypanum herreri*, *Trypanosoma vivax* (Liem strain), *Trypanosoma copemani* and *Trypanosoma cruzi* (Silvio strain) were not available from the sequence reads for subsequent analyses. Additionally, REPuter was also used to identify repeat sequences including direct and palindromic repeats within the divergent region of these 42 trypanosomatid species [41]. A minimum repeat size of 60 bp and 15 bp was chosen respectively for each repeat identification. Tandem repeats in all five species were identified using Tandem Repeats Finder version 4.09 with default settings [42].

### 2.4. MSA Phylogenetic Analysis

A phylogenetic analysis was performed using the entire coding region from the maxicircle of 46 trypanosomatid species to investigate the evolutionary relationships between members of the Trypansomatidae. All sequences were aligned using the MUSCLE algorithm implemented in the Seaview software package [43]. Phylogenetic relationships were inferred using the maximum likelihood optimality criterion using PhyML version 3.0. For ML trees, the best-fit model of evolution, GTR+I+G was selected using jModelTest 2.1 under the Bayesian information criterion [44]. Bootstrap support for clade topologies was estimated following the analysis of 1000 pseudo-replicate datasets using a heuristic tree search.

### 2.5. Alignment-Free FFP Analysis

The alignment-free FFP analysis was performed on the maxicircle coding region genome of 46 trypanosomatid species using the command line software FFP version 3.1.9 [35]. To determine the optimal length of k-mer, the FFPvprof utility was used to determine the average lower word length limit and the FFPvreprof utlity was used to determine the average upper word limit of all 46 trypanosomatid species [20]. The optimum k-mer length can be identified from the overlapping region of length ranges (i.e., average of the upper and lower length limit). To determine the lower k- mer length limit, FFPvprof calculates the number of k-mers (and length) that occur at least twice between the dataset. To determine the upper k-mer length limit, FFPreprof calculates the relative entropy frequency (REF) between the expected and observed frequencies of specified k-mer lengths. Following optimisation, the UNIX-style command line pipeline was used to generate a distance matrix, which is used for the subsequent phylogenomic inference using the program Phylip version 3.697 [45]. Bootstrap support for clade topologies was estimated using the FFPboot utility following the analysis of 1000 pseudo-replicate datasets.

### 2.6. Estimating Divergence Time

Divergence dates of the trypanosomatid family were estimated using the Realtime method [1] and the General Time Reversible model [2] of the MEGA7 package. The maximum likelihood of this timetree was computed using one calibration constraint; the divergence of *Leishmania enriettii* and *Leishmania macropodum* approximately 40 MYA [46,47]. The maxicircle sequence of the monoxenous trypanosomatid *Paratrypanosoma confusum* served as an outgroup.

## 3. Results

### 3.1. Assembly of Data from Illumina and Hybrid Illumina/Pacbio Reads

The initial assemblies of Illumina paired-end reads using the SPAdes assembler resulted in the generation of twelve maxicircle genomes from the trypanosomatid family. Of these, we used the same Illumina dataset of eight species; *L. aethiopica*, *L. amazonensis*, *L. braziliensis*, *L. guyanensis*, *L. infantum*, *L. mexicana*, *L. tropica* and *T*. *brucei rhodesiense* in addition to their corresponding PacBio data for a hybrid assembly. The assemblies from both short-read and combination short- and long-read data resulted in a high-quality assembly of the maxicircle kDNA. A comparison of the final contig generated from paired-end reads to the hybrid revealed a 100% sequence identity between the coding region, reflecting the excellent accuracy of the maxicircle genome assembled from whole gene sequence generated by both SGS and TGS (Table 1). Comparison of the non-coding divergent region revealed a sequence identity >99% between the contigs generated from paired-end reads to the hybrid. The long-read lengths of the PacBio data were extremely valuable for the de novo assembly of the maxicircle, allowing us to overcome the problems caused by the repetitive nature of the divergent region. The difference in sequence identity of the divergent region is due to the increased resolution of the hybrid approach, yielding a more accurate estimate of the length of the divergent region than that of Illumina reads alone. Compared to that of the hybrid approach, SGS reads of *L. aethiopica*, *L. amazonensis*, *L. braziliensis*, *L. guyanensis, L. infantum*, *L. mexicana, L. tropica* and *T. brucei rhodesiense* assembled only 20%, 11%, 20%, 43%, 49%, 46%, 96% and 88% of the divergent region produced from TGS respectively.

### 3.2. Genomic Organisation and Patterns of the Maxicircle

**Figure 1:**
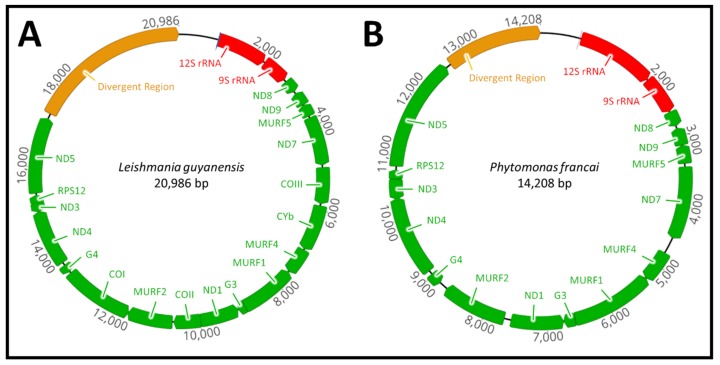
Graphical map of *Leishmania guyanensis* and *Phytomonas françai* maxicircle genomes assembled from Illumina and PacBio sequencing. Comparison of the maxicircle genome of (**A**) *Leishmania guyanensis* and (**B**) *Phytomonas françai*. *Phytomonas* spp. is characterised by the loss of the three subunits of the cytochrome *c* oxidase (COI, COII and COIII) and single subunit of cytochrome *b* (CYb).

Sequence analysis of the maxicircle from representative species of the genera *Leishmania*, *Porcisia*, *Endotrypanum*, *Zelonia*, *Leptomonas*, *Crithidia*, *Herpetomonas*, *Angomonas*, *Blechomonas*, *Trypanosoma* and *Paratrypanosoma* revealed a conserved region typical for the maxicircle. The conserved region includes; 12S rRNA (large subunit), 9S rRNA (small subunit), seven subunits of NADH dehydrogenase, (ND8, ND9, ND7, ND1, ND3, ND5 and ND5), three subunits of cytochrome *c* oxidase (COI, COII and COIII), one subunit of the cytochrome *bc*_1_ complex (CYb) a single ribosomal protein (RPS12) and four open-reading frames whose role is unknown (MURF1, MURF2, MURF4 and MURF5). There was also two G-rich (G3 and G4) or C-rich (CR3 and CR4) pan-edited cryptogenes in *Leishmania* and *Trypanosoma* species respectively. Analysis of the *Phytomonas françai* maxicircle revealed that the genes for subunits I, II and III (COI, COII and COIII) of cytochrome *c* oxidase and cytochrome *bc_1_* complex (CYb) were missing (Figure 1).

**Figure 2:**
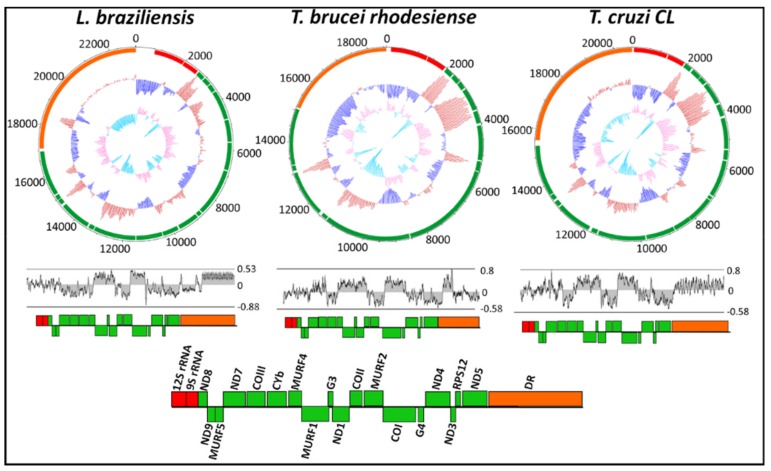
Circular confirmation of GC plot and GC skew of the maxicircle kinetoplast for *Leishmania braziliensis, Trypanosoma brucei rhodesiense and Trypanosoma cruzi.* The outer ring indicates gene arrangement and gene distribution. The middle circle represents the GC plot showing GC% content (dark blue for below-average and red for above-average) and the inner circle represents the GC skew (light blue for positive and orange for negative). The AT skew and corresponding gene arrangement and distribution of each species is shown below the circular plot.

The overall nucleotide frequency and skew throughout the maxicircle genome of all 46 trypanosomatid species is shown in Supplementary Materials S2. The overall AT-richness of the maxicircle ranged from 58–82%, reflecting the AT-overabundance of the mitochondrial genome as a whole. Of the clinically important *L. braziliensis*, *T. brucei rhodesiense* and *T. cruzi* (*CL* strain), the GC skew and GC% content is represented in the top panel of Figure 2. From the GC content, it was observed that the AT-richness of *T. brucei rhodesiense* and *T. cruzi* is primarily due to the repeat region, where the GC% of the divergent region is predominately below average (dark blue lines). The AT-richness of *L. braziliensis* is still high (80%), although the GC plot demonstrates that the AT-richness in the divergent region is to a lesser extent less than that of *T. brucei rhodesiense* and *T. cruzi*, with the AT-abundance seen throughout the entire genome. The AT skew is shown in the bottom panel of Figure 2. The AT skew of all three species demonstrates the bias towards a T-rich sequence that is more pronounced in the coding region compared to that of the DR, which demonstrates a bias towards an A-rich nucleotide frequency.

**Figure 3:**
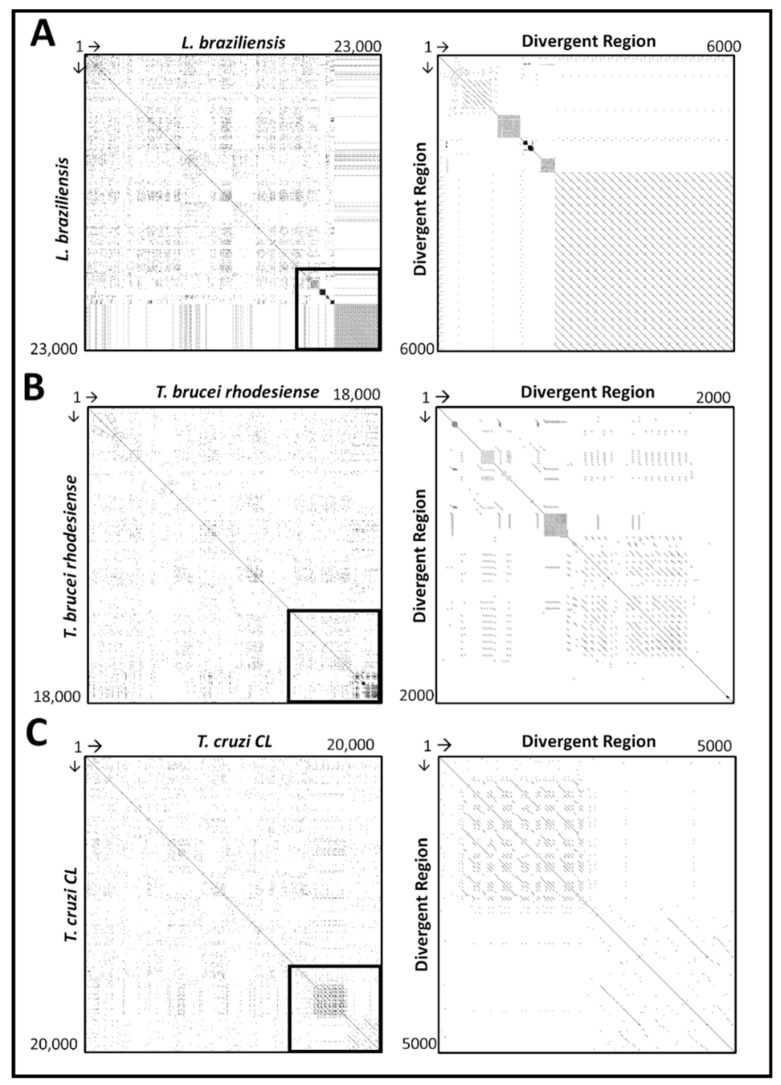
Self Dottup plot comparative analysis of the entire maxicircle genome (right panel) and divergent region (left panel) of *Leishmania braziliensis* (**A**), *Trypanosoma brucei rhodesiense* (**B**) and *Trypanosoma cruzi* (**C**) against their own sequences. The sequence of each species is placed on the axis and full identity over a 10 bp-long window is represented by a dot. The main diagonal line represents the sequence’s alignment with itself and the lines about the main diagonal represent repetitive patterns within the maxicircle sequence.

A dot matrix search of the entire maxicircle sequence (left panel) and divergent region (right panel) of *L. braziliensis*, *T. brucei rhodesiense* and *T. cruzi* (*CL* strain), versus itself is shown in Figure 3. The comparative analysis for the remaining trypanosomatid species can be found in Supplementary Materials S3. Based on the left panel of each graph, we can see a clear distinction between the coding and non-coding region of the maxicircle sequence, which is highlighted in a black outline. The box structures located on and residing symmetrically around the line of identity are indicative of a clustered organisation of repetitive sequences. Multiple diagonal lines seen in all three panels are indicative of direct repeats. The length of the divergent region of the various trypanosomatid species is significantly variable and thus responsible for the size difference of the complete maxicircle genome of the kinetoplast.

**Figure 4:**
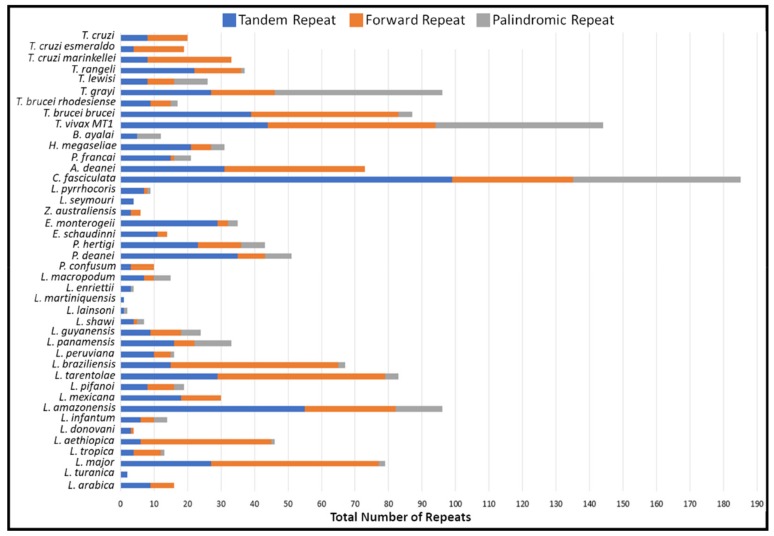
Analysis of total repeated sequences in the maxicircle divergent region of various trypanosomatid species. Totals of three repeat types; (Blue) Number of tandem repeats; (Orange) Number of forward repeats; (Grey) Number of palindromic repeats.

The repeat analysis demonstrated that a different number of the various repeats were observed in the species compared (Figure 4). In these divergent region sequences, the repeat analysis detected tandem, palindromic, forward repeats. The various repeats found within the non-coding region differed significantly between species (Supplementary Materials S4). Following suit with the overall composition of the maxicircle, the forward, tandem and palindromic repeats all demonstrated a strong bias towards an AT-rich repeat in all trypanosomatid species compared. Tandem repeats identified were highly abundant and densely interspersed throughout the divergent region. The majority of tandem repeats are arranged in clusters composed of predominately short A_n_T_n_ repeats.

### 3.3. Phylogenetic Analyses

**Figure 5:**
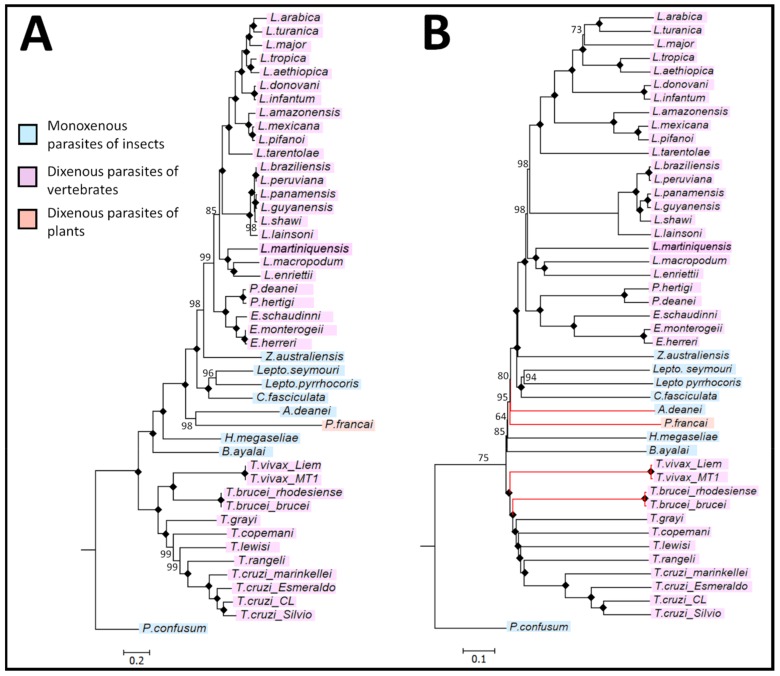
Inferred evolutionary relationships between species of the trypanosomatid family using aligned and alignment-free analysis of the maxicircle coding region. (**A**) PhyML-derived phylogeny showing the relationships between members of the trypanosomatid family using a multiple sequence alignment analysis. The maximum likelihood optimality criterion was used for the phylogenetic inference of the dataset with 1000 bootstrap samples. (**B**) Featue frequency profiles (FPP)-estimated phylogeny between species of the trypanosomatid family using an alignment-free analysis of the maxicircle coding region. Branches in red represent species whose clustering differs from those in (A). In both (A) and (B) a black diamond highlights a node that obtained a bootstrap value of 100% confidence and the scale bars depict the number of nucleotide substitutions per position.

To assess how FFP-based phylogenies compares to the traditionally aligned-phylogenies, the phylogenetic relationships showing the genetic distance between members of the Trypanosomatidae based on the MSA- and AF-approach is shown in Figure 5A,B. The sequences of 46 trypanosomatid species were used to determine the optimal word length or k-mer for subsequent alignment-free analyses. From the FFPvprof and FFPreprof analyses, the average lower world length (k-mer) limit of the 45 species was 14 (*k* = 14) and the average upper world length limit was 30 (*k* = 30) leading to the selection of k-mer lengths of 22 (Supplementary Materials S5).

In the inferred phylogenies, all *Leishmania* and *Trypanosoma* species formed two separate, strongly supported monophyletic clades. The FFP analysis showed the same subgrouping of the Trypanosomatidae, with all six subfamilies and eleven genera corresponding to the PhyML-generated phylogenetic tree. The subfamily Leishmaniinae unites the monoxenous protozoans of insects (genera *Zelonia*, *Leptomonas* and *Crithidia*) and dixenous protozoans of insects and vertebrates (genera *Leishmania*, *Endotrypanum* and *Porcisia*). The monoxenous subfamilies Phytomonadinae, Strigononadinae and Blechomonadinae include parasites of the genera *Herpetomonas*, *Angomonas* and *Blechomonas* respectively. In agreement with a recent study [11], despite *T. rangeli* and *T. lewisi* belonging to the same subgenus *Herpetosoma*, *T. rangeli* is more closely related to *T. cruzi*, clustering with the Schizotrypanum clade of trypanosomes.

The discrepancies observed were the branching of the Salivarian trypanosomes, where *T*. *brucei brucei* and *T*. *brucei rhodesiense* formed a clade basal to the *T*. *vivax* clade and between the monoxenous *A. deanei and P. françai*. The distance matrix generated from the alignment-free analysis is listed in Supplementary Materials S6.

### 3.4. Divergence Time Estimates of the Trypanosomatidae

**Figure 6:**
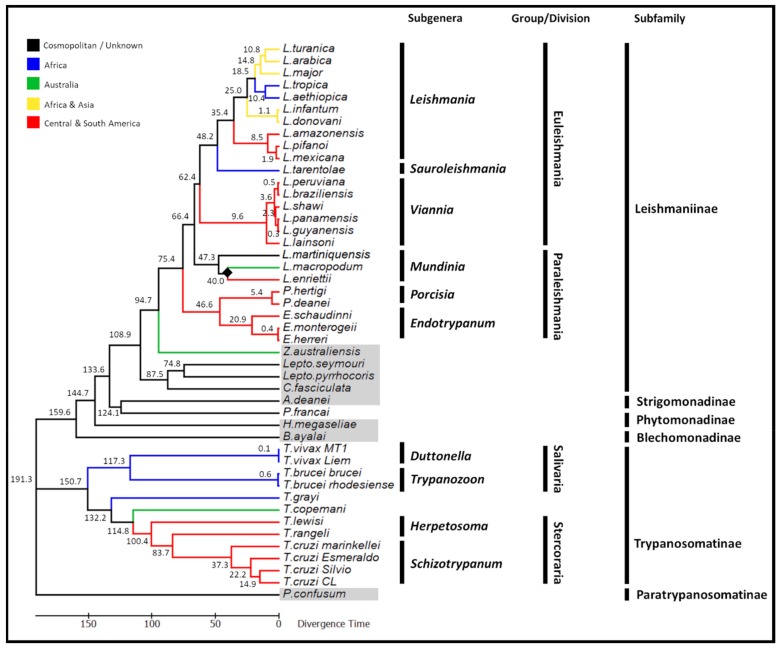
Phylogenetic time tree demonstrating the complex evolutionary relationships of the trypanosomatid family using the coding region of the maxicircle kDNA. The maximum likelihood of this tree was inferred using the GTR model. The time tree was calculated using a single calibration date, illustrated by a solid black diamond (the separation of *Leishmania macropodum* and *Leishmania enriettii* approximately 40 million years ago). Predicted divergence times of the various species are displayed on the nodes of the tree. Monoxenous trypanosomatid species are highlighted in grey.

The node depicting the separation of *L. macropodum* and *L. enriettii* was chosen as a calibration marker. The divergence date was established at an average of 40 MYA, which is the time period when Australia and South America became separated, representing a minimum date of divergence [46,47]. From this calibration point, a common ancestor to the Trypanosomatinae subfamily and specifically the *T. cruzi* clade (*T. cruzi* and *T. rangeli*) was predicted to have appeared 150 MYA and 84 MYA respectively (Figure 6). The dixenous members of the Leishmaniinae was predicted to have first appeared 95 MYA, arising from a monoxenous ancestor.

## 4. Discussion

In this study we applied the alignment-free analysis of frequency feature profiles (FFP) for the phylogenomic analysis of the trypanosomatid family based on the entire maxicircle coding region genome. For closely related organisms i.e., within a single genus, alignment-based methods are preferred because in the most part, their genetic similarities allow a relatively straight-forward alignment of high-quality [33]. However, in the context of looking at an entire family of organisms (such as the Trypanosomatidae), the various members exhibit greater variation than species within a single genus. Knowledge of these evolutionary processes demonstrates that there is going to be genetic discrepancies (i.e., the loss of maxicircle genes in *Phytomonas* spp.) which is going to negatively impact on the quality of any sequence alignment. Due to these less conserved sequences and scale of the dataset (greater than 15 kb across 46 species), the alignment-free method is preferred for the analysis of distant relatives, allowing the analysis of organisms which exhibit greater variation in the number and order of genetic elements.

In accordance with published standards in the assessment of robustness (i.e., bootstrapping), the percentile method validates the accuracy of a node, with a confidence value of >60% in support of the observed clade [48]. The MSA and AF trees showed extremely similar and robust topologies (Figure 5), demonstrating the suitability of the alignment-free FFP method for phylogenetic analyses of the Trypanosomatidae. All major clades (*Leishmania*, *Endotrypanum*, *Porcisia*, *Zelonia*, *Leptomonas*, *Crithidia*, *Herpetomonas*, *Phytomonas*, *Angomonas*, *Blechomonas*, *Trypanosoma* and *Paratrypanosoma,* cluster with their respective subgenera within the trypanosomatid family with high bootstrap confidence. Analysing a large dataset from an entire family of parasites, a greater divergence is expected than a typical phylogenetic analysis of a few, closely related genera, which can impact on the accuracy of the sequence alignment. In addition, the overall running times of alignment-free phylogenetic inference were shorter (run time less than one day compared to >3 days for alignment and PhyML analysis), making it an attractive approach for large datasets >15 kb. However, important issues need to be taken into consideration for AF-approaches, namely that optimal k-mer lengths must be determined to establish accurate phylogenomic relationships between related organisms. Nevertheless, having the advantage of not requiring pre-alignment of a large collection of maxicircle genome sequences, FFP analysis allowed the rapid, accurate and robust phylogenetic analysis of a large group of diverse species within the Trypanosomatidae.

Kinetoplastid parasites of the trypanosomatid family possess a unique, single, densely-packed periflagellar network of DNA circles known as the kinetoplast [12,49]. In our analysis, both paired- end short reads and a hybrid protocol using short- and long-read data resulted in the high- quality assembly of the maxicircle genome (Table 1). The maxicircle sequences generated from SGS and hybrid assemblies resulted in 100% identity between the coding region and >99% identity between the divergent region (due to the difference in sequence length of the divergent region between Illumina and hybrid assemblies) of each respective species. The hybrid protocol offered an alternative approach, capable of overcoming the difficulties present in assembling complex genomic regions from short sequencing reads. Our hybrid assemblies resulted in the resolution of the divergent region (DR), generating highly contiguous, accurate assemblies of the DR, even when these regions contain a large number of near-identical repeats.

Analysis of the repetitive sequences demonstrated that the various trypanosomatid species have a variable number of repeat arrays throughout their divergent region (Figure 4 and Supplementary Materials S4). The non-coding divergent region of the maxicircle remains the most poorly studied region of kinetoplast DNA [16,50,51]. From our analysis, we can see that the overall DR structure is composed almost entirely of various repeat arrays, with a clear distinction between the coding-region of the maxicircle kDNA (Figure 3 and Figure 4). The high repeat content of the divergent region posed a substantial obstacle to the assembly and output length of the maxicircle kDNA with Illumina short- reads alone. Compared to that of the hybrid approach, SGS reads of *L. aethiopica*, *L. amazonensis*, *L. braziliensis*, *L. guyanensis*, *L. infantum*, *L. mexicana*, *L. tropica* and *T. brucei rhodesiense* assembled only 20%, 11%, 20%, 43%, 49%, 46%, 96% and 88% of the divergent region produced from TGS respectively.

Protozoan mitochondrial genomes often display higher AT% than mitochondrial genomes from metazoans [12,52]. Thus, it is expected that in the mitochondrial homologue in trypanosomatids; the kinetoplast will have a low GC%. The extreme overabundance of AT% in the maxicircle genome of trypanosomatids is primarily due to the repetitive portion of the non-coding divergent region (Figure 2). The AT skew demonstrates the bias towards an AT-rich maxicircle genome of trypanosomatid species.

The vast majority of maxicircles in the trypanosomatid family contain the same set of genes and genomic organisation pattern. The notable exception to this is the plant parasite *Phytomonas françai*, which lacks the respiratory chain complexes III and IV, including the three subunits of the cytochrome *c* oxidase (COI, COII and COIII) and single cytochrome *b* (CYb), respectively (Figure 1). The respiratory chain complexes III and IV are required to maintain a complete electron transport chain in trypanosomatid parasites, playing a critical role in the biochemical production and synthesis of adenosine triphosphate (ATP) [53,54]. It is speculated that the absence of these subunits is related to the adaptation of *Phytomonas* spp. to the carbohydrate-rich medium of the host plant environment [55]. In the presence of this carbohydrate-rich medium, the cytochrome-mediated respiration complexes of *Phytomonas* were lost after their function became nonessential and the presence of the glycolysis metabolic pathway in glycosomes alone was sufficient for ATP production [55,56]. The feeding behaviour of the insect host is also speculated to have played a role in the loss of cytochromes in *Phytomonas* [57]. Phytophagous hemipteran insects feed exclusively on the sap and juices of plants that are rich in carbohydrates. This suggests a passive, mechanical transmission from plant to insect host that does not require a metabolic shift from a carbohydrate to an amino acid metabolism [58]. Previous studies have also demonstrated the absence of these respective subunits in *Phytomonas serpens* and *Phytomonas* sp. HART1, demonstrating this respiratory deficiency is characteristic of these dixenous plant trypanosomatids [55,57,58].

A second notable exception is the mammalian infecting *Trypanosoma brucei evansi* and *Trypanosoma brucei equiperdum* [59]. Originally considered separate species due to their differences in host-range, transmission and pathogenicity, recent data demonstrates that *T. b. evansi* and *T. b. equiperdum* represent dyskinetoplastic subspecies of *T. brucei,* characterized by the complete or partial loss of their maxicircle kDNA, although their taxonomic status is often debated [60,61,62]. Recent studies have shown that mutations located in the nuclear-bound ATPase subunit γ of some *T. b. evansi* and *T. b. equiperdum* were found to compensate for the total or partial absence of maxicircle kDNA, demonstrating an important nuclear/kinetoplast interaction for the viability of these species [61].

The relationships between the monoxenous trypanosomatids and their dixenous relatives have been extensively debated over the last few decades [63,64]. It was speculated that dixenous parasitism of the Trypanosomatidae has independently evolved several times over the course of history from the monoxenous ancestors, giving rise to the *Trypanosoma*, *Leishmania*, *Endotrypanum*, *Porcisia* and *Phytomonas* lineages [63]. As such, it is impossible to answer questions surrounding the origins of the trypanosomatid family without studying the non-pathogenic, monoxenous relatives, which is reflected by the rising number of papers published in this field [46,65,66,67,68,69]. From the molecular data, several major clades can be identified including; *Leishmania*, *Endotrypanum*, *Porcisia*, *Zelonia*, *Leptomonas*, *Crithidia*, *Herpetomonas*, *Angomonas*, *Blechomonas*, *Trypanosoma* and *Paratrypanosoma*. All clades generated support a recent proposal on the classification of the trypanosomatids, specifically the taxonomic validity of the *Endotrypanum*/*Porcisia* genera and the establishment of the subfamily Trypanosomatinae to encompass species of the trypanosomes [5,70]. Molecular clock analyses suggest that at least three lineages independently acquired the ability to infect two hosts (i.e., dixenous parasitism) including plants (*Phytomonas* spp.) and vertebrates (*Trypanosoma* and *Leishmania*/*Porcisia*/*Endotrypanum*) approximately 133, 190 and 95 MYA respectively. Thus, our analyses provide strong support for the multiple and independent origins of the dixenous life-style of the trypanosomatids.

The supercontinents origin of the dixenous Leishmaniinae, where the dixenous members first emerged from monoxenous ancestors during the continental separation of Gondwana is now widely accepted [46,47,63]. As suggested by the supercontinents hypothesis of dixenous parasitism, the earliest dixenous members of the Leishmaniinae first emerged in the late Cretaceous period between 77–140 MYA, during the predicted breakup of Gondwana [19]. Based on our phylogenetic analysis, the dixenous genera *Leishmania*, *Endotrypanum* and *Porcisia* emerged as distinct monophyletic lineages from a common monoxenous ancestor approximately 95 million years ago (Figure 6). Detailed extensively in our previous work [19], in summary the first emergence of dixenous parasitism within the Leishmaniinae subfamily coincides with when the radiation of mammals first began during the Cretaceous period [71].

The phylogenetic analysis of the trypanosomatid family supported the monophyletic lineage of the trypanosomes, having evolved from a monoxenous ancestor with high statistical support (Figure 5). Trypanosomes found in mammals (and humans) are grouped into two sections: Stercoraria (including members of the subgenera *Herpetosoma* and *Schizotrypanum*), which develops in the posterior portion of the insect host digestive tract and Salivaria (including members of the subgenera *Duttonella* and *Trypanozoon*), which develops in the anterior region of the insect digestive tract [58]. Previous analyses proposed an early divergence of salivarian trypanosomes, which involved the ancient split of the trypanosomes into one clade containing all salivarian species and the other branch containing all non-salivarian lineages [72,73,74]. Based on the SSU rRNA, it was proposed that the emergence of the salivarian lineage first appeared approximately 300 MYA, although the authors were cautious about these date estimates due to the use of the SSU rRNA as the taxonomic marker [72]. Consistent with an ancient salivarian divergence, molecular clock analyses based on the maxicircle phylogenies suggest that the salivarian trypanosomes separated from other trypanosomes approximately 150 MYA (Figure 6). It is speculated that early African trypanosomes were most likely gut parasites or commensals of early insects, however the appearance of tsetse flies approximately 35 MYA facilitated the transmission to mammals by these blood-feeding insects [75]. In addition, the use of the maxicircle coding region as a taxonomic marker also provided strong support for the placement of *Trypanosoma vivax* with other African salivarian trypanosomes including *Trypanosoma brucei* [64].

The analysis of the coding region of the maxicircle also sheds light on the evolution of the *T. cruzi* clade, which encompasses most African, American and European trypanosomes from bats and terrestrial mammals [74]. In recent years, the southern super-continent trypanosome hypothesis has come under scrutiny, with some suggesting it can no longer be considered correct that *T. cruzi* first emerged in the New World and *T. brucei* in the Old World following the continental split of Africa and South America 100 MYA [47,76,77,78]. The new theory gaining the greatest support proposes that the *T. cruzi* clade (*T. cruzi* and *T. rangeli*) evolved more recently than originally thought within a broader clade of bat trypanosomes [73]. Our analysis suggests that the common ancestor of the *T. cruzi* clade first appeared approximately 84 million years ago. This timeframe provides strong additional support for the ‘bat-seeding’ hypothesis, suggesting that the common ancestor of the *T. cruzi* clade is likely to have evolved following the diversification of bats approximately 70–58 million years ago [79,80]. Using a geological time point i.e., the divergence of *L. enriettii* and *L. macropodum* as the calibration date facilitates suggestions on the emergence of the *T. cruzi* clade independently of the diversification of bats. This is an important advancement for the ‘bat-seeding’ hypothesis, previously not feasible in the absence of WGS data for *L. macropodum,* as it eliminates any bias or skew that may be introduced through using the estimated time of divergence of the host species (i.e., a secondary calibration) [80].

## 5. Conclusions

In conclusion, the use of the entire coding region of the maxicircle kinetoplast DNA in alignment-free analyses provided an exceptionally robust evolutionary insight into the relationships and patterns within the trypanosomatid family. We believe the present study demonstrates the applicability of AF-based approaches such as FFP to produce reliable and robust phylogenomic analyses to establish and summarise the current evolutionary relationships of the Trypanosomatidae. We suggest that future researchers aiming to analyse this diverse and widespread relationship should consider using this described approach to deal with the ever-increasing amount of sequence data that are becoming available. Ultimately, knowledge of these deep-rooted lineages is exceptionally useful for future analysis involving reconstruction of any evolutionary scenario involving these flagellated protozoans.

## Figures and Tables

**Table 1:** Comparison of the short-read Illumina and hybrid Illumina/PacBio assembly of various trypanosomatid species showing output length and sequence identity (%).

	Coding Region		Non-Coding Region		Overall	
	Size (bp)	% Identity	Size (bp)	% Identity	Size (bp)	% Identity
*L. aethiopica* (Illumina)	16 210	100	304	100	17 264	100
*L. aethiopica* (Hybrid)	16 210		1552		18 571	
*L. amazonensis* (Illumina)	16 381	100	527	99.7	17 941	100
*L. amazonensis* (Hybrid)	16 381		4621		23 616	
*L. braziliensis* (Illumina)	16 232	100	1121	98.5	18 118	99.9
*L. braziliensis* (Hybrid)	16 232		5745		23 012	
*L. guyanensis* (Illumina)	16 235	100	1607	100	18 753	100
*L. guyanensis* (Hybrid)	16 235		3694		20 986	
*L. infantum* (Illumina)	16 277	100	628	99.5	18 287	100
*L. infantum* (Hybrid)	16 277		1280		18 637	
*L. mexicana* (Illumina)	16 472	100	736	99.7	17 946	100
*L. mexicana* (Hybrid)	16 472		1606		18 696	
*L. tropica* (Illumina)	16 229	100	1582	97	18 800	99.5
*L. tropica* (Hybrid)	16 229		1644		19 020	
*T. brucei rhodesiense* (Illumina)	14 905	100	2785	97	18 200	99.5
*T. brucei rhodesiense* (Hybrid)	14 905		3131		18 583	

## Supplementary Materials

The following are available online at https://www.mdpi.com/2076-0817/8/3/157/s1, Table S1: List of trypanosomatid species used in this study. Table S2: The overall nucleotide frequency and skew throughout the maxicircle genome of various trypanosomatid species. Figure S3: Self Dottup plot comparative analysis of the entire maxicircle genome (right panel) and divergent region (left panel) of various trypanosomatid species. Figure S4: Analysis of repeated sequences in the maxicircle divergent region. Figure S5: Determination of optimal feature/k-mer length (k) for 46 trypanosomatid species. Table S6: Comparison of distance matrices showing genetic differences between maxicircle kDNA of various trypanosomatid species. Below diagonal: genetic distance calculated from multiple sequence-alignment method, above diagonal: genetic distance calculated from alignment-free FFP method.
