# Supplementary material for: Evolutionary Insight into the Trypanosomatidae Using Alignment-Free Phylogenomics of the Kinetoplast
Source: Pathogens. 2019 Sep 18;8(3):157. doi: 10.3390/pathogens8030157 (PMC6789588; doi:10.3390/pathogens8030157)

Evolutionary insight into the Trypanosomatidae using alignment-free phylogenomics of the kinetoplast

Alexa Kaufer [1,*], Damien Stark [2] and John Ellis [1]

Supplementary Materials S1 – S5

S1 – Table of all trypanosomatid species used in this study

*Sequences assembled in the study

S2 -Table of the overall nucleotide frequency and skew throughout the maxicircle genome of various trypanosomatid species.

S3 - Self dottup plot comparative analysis of the entire maxicircle genome (right panel) and divergent region (left panel) of various trypanosomatid species

S4 - Analysis of repeated sequences in the maxicircle divergent region

S5 - Determination of optimal feature/k-mer length (*k*) for 46 trypanosomatid species.

(A) Graph shows the determination of the minimum feature/k-mer length for DNA. The optimal lower-feature length (*k*) is considered the point where the most features can be found in most genomes (i.e. peak of curve). (B) Graph shows the determination of the maximum feature/k-mer length for DNA. The optimal upper-feature length (*k*) is considered the point where the genome curves start having zero REF or begins falling to <10% of their REF maximum values [24].

S1 – Table of all trypanosomatid species used in this study.

Species	Genbank/TriTryp	Species	Genbank/TriTryp
A. deanei	KJ778684	*L. pyrrhocoris*	BK010873
B. ayalai	Baya_253	*L. seymouri*	Lsey_0394
C. fasciculata	CfaCI_maxi	*L. shawi*	BK010883
E. herreri	MK514112	*L. tarentolae*	MK514114
E. monterogeii	EMOLV88	*L. tropica*	MK514115*
E. schaudinni	BK010874	*L. turanica*	BK010887
H. megaseliae	ERX922862	*P. confusum*	SRX3339697
L. aethiopica	BK010882*	*P. deanei*	BK010886
L. amazonensis	SRX5187732*	*P. hertigi*	BK010885
L. arabica	BK010878	*T. brucei_brucei*	M94286.1
L. braziliensis	MK514111*	*T. brucei_rhodesiense*	SRX3199071*
L. donovani	CP022652.1	*T. copemani*	MG948557
L. enriettii	BK010880	*T. cruzi_CL*	DQ343645.1
L. guyanensis	BK010876*	*T. cruzi_Esmeraldo*	DQ343646
L. infantum	BK010877*	*T. cruzi_Marinkellei*	KC427240
L. lainsoni	BK010879	*T. cruzi_Silvio*	FJ203996
L. macropodum	SRX5006815*	*T. grayi*	SRX620256*
L. major	MK514113	*T. lewisi*	KR072974
L. martiniquensis	SRX5006816*	*T. rangeli*	KJ803830
L. mexicana	SRX5187730*	*T. vivax_Liem*	KM386509
L. panamensis	BK010875	*T. vivax_MTI*	KM386508
L. peruviana	BK010881	*Z. australiensis*	MK514117
L. pifanoi	BK010884	*P. francai*	SRX2165265*

S2. The overall nucleotide frequency and skew throughout the maxicircle genome of various trypanosomatid species.

	**Ltur**	**Lara**	**Lmaj**	**Ltro**	**Laet**	**Linf**	**Ldon**	**Lmex**	**Lpif**	**Lama**	**Ltar**	**Lguy**	**Lpan**	**Lsha**	**Lbra**
% A	33	37	33	27	27	35	44	24	35	39	38	35	37	36	40
% C	10	7	10	36	8	9	12	10	10	9	9	8	8	8	9
% G	14	11	14	6	17	13	9	20	12	11	12	12	12	12	11
% T	43	45	43	31	48	43	35	46	43	41	41	45	43	44	40
% A + T	76	82	76	58	75	78	19	70	78	80	79	80	80	80	80
% G + C	24	18	24	42	25	22	21	30	22	20	21	20	20	20	20
AT skew	-0.13	-0.09	-0.13	-0.07	-0.28	-0.10	0.11	-0.31	-0.10	-0.03	-0.04	-0.13	-0.08	-0.1	0
GC skew	0.17	0.22	0.17	-0.71	0.36	0.18	-0.14	0.33	0.09	0.10	0.14	0.2	0.2	0.2	0.1

	Lper	Llai	Lmar	Lmac	Lenr	Pher	Pdea	Esch	Emon	Eher	Zaus	Lsey	Lpyr	Cfas	Hmeg
% A	36	36	34	34	35	38	39	37	38	36	33	34	34	35	38
% C	8	9	9	10	10	10	11	10	9	10	12	11	12	12	13
% G	12	13	14	14	13	13	13	12	12	13	15	13	14	13	14
% T	44	42	43	42	42	39	37	41	41	41	40	42	40	40	35
% A + T	80	78	77	76	77	77	76	78	79	77	73	76	74	75	73
% G + C	20	22	23	24	23	23	24	22	21	23	27	24	26	25	27
AT skew	-0.1	-0.08	-0.11	-0.10	-0.09	-0.01	0.02	-0.05	-0.04	-0.06	-0.09	-0.11	-0.08	-0.06	0.04
GC skew	0.2	0.18	0.21	0.17	0.13	0.13	0.08	0.09	0.14	0.13	0.11	0.08	0.08	0.04	0.04

	Adea	Baya	Tvmt	Tvli	Tbru	Tgra	Tcop	Tlew	Tran	Tcma	Tces	Tcsi	Tccl	Pcon
% A	34	41	38	38	42	39	37	41	45	39	40	27	39	36
% C	16	9	10	10	9	12	13	12	13	10	11	9	11	13
% G	15	13	12	13	14	13	15	12	11	13	13	14	14	15
% T	35	37	40	39	35	36	35	35	31	38	36	50	36	36
% A + T	69	78	78	77	77	75	72	76	76	77	76	77	75	72
% G + C	31	22	22	23	23	25	28	24	24	23	24	23	25	28
AT skew	-0.01	0.05	-0.02	-0.01	0.09	0.04	0.03	0.08	0.18	0.01	0.05	0.30	0.04	0
GC skew	-0.03	0.18	0.09	0.13	0.22	0.04	0.07	0	-0.08	0.13	0.08	0.22	75	0.07

Abbreviated species: Ltur (*L. turanica*); Lara (*L. arabica*); Lmaj (*L. major*); Ltro (*L. tropica*); Laet (*L. aethiopica*); Linf (*L. infantum*); Ldon (L. *donovani*); Lmex (*L. mexicana*); Lpif (*L. pifanoi*); Lama (*L. amazonensis*); Ltar (*L. tarentolae*); Lguy (*L. guyanensis*); Lpan (*L. panamensis*); Lsha (L. shawi); Lbra (*L. braziliensis*); Lper (*L. peruviana*); Llai (*L. lainsoni*); Lmar (*L. martiniquensis*); Lmac (*L. macropodum*); Lenr (*L. enriettii*); Pher (*P. hertigi*); Pdea (*P. deanei*); Esch (*E. schaudinni*); Emon (*E. monterogeii*); Eher (*E. herreri*); Zaus (*Z. australiensis*); Lsey (*L. seymouri*); Lpyr (*L. pyrrhocoris*); Cfas (*C. fasciculata*); Hmeg (*H. megaseliae*); Adea (*A. deanei*); Baya (*B. ayalai*); Tvmt (*T. vivax MT1*); Tvli (*T. vivax Liem*); Tbru (*T. brucei brucei*); Tgra (*T. grayi*); Tcop (*T. copemani*); Tlew (*T. lewisi*); Tran (*T. rangeli*); Tcma (*T. cruzi marinkellei*); Tces (*T. cruzi esmeraldo*); Tcsi (*T. cruzi Silvio*); Tccl (*T. cruzi CL*); Pcon (*P. confusum*)

S3 - Self dottup plot comparative analysis of the entire maxicircle genome (right panel) and divergent region (left panel) of various trypanosomatid species

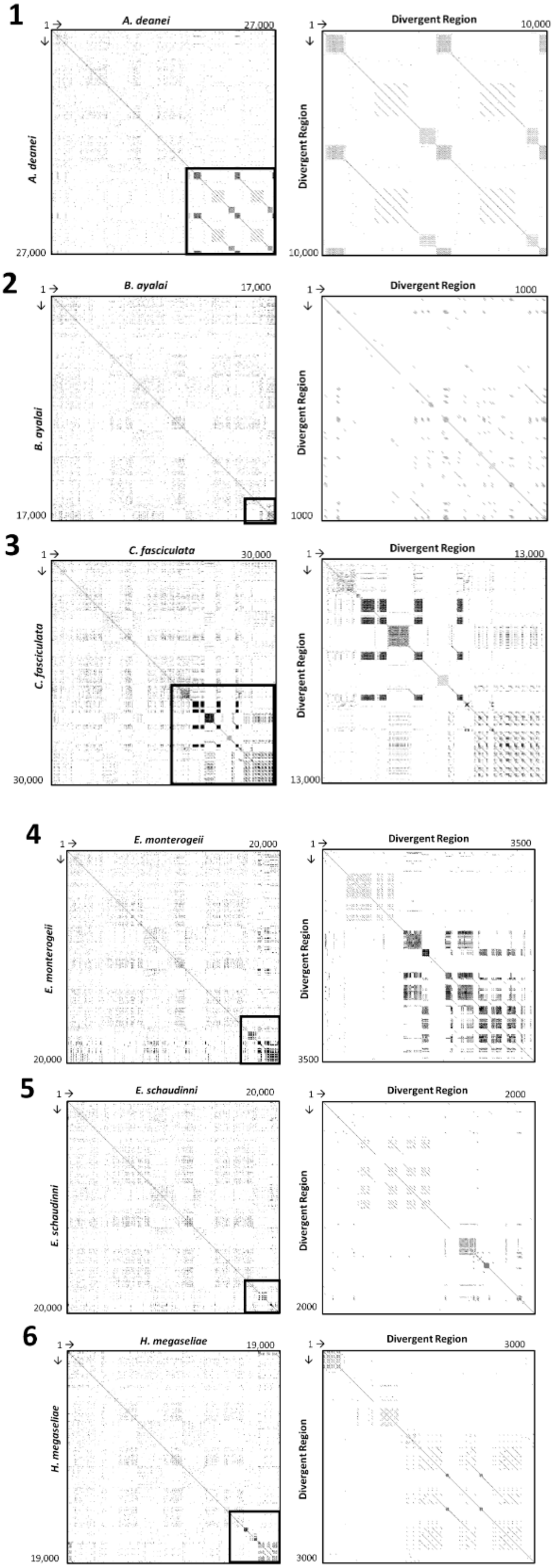

7

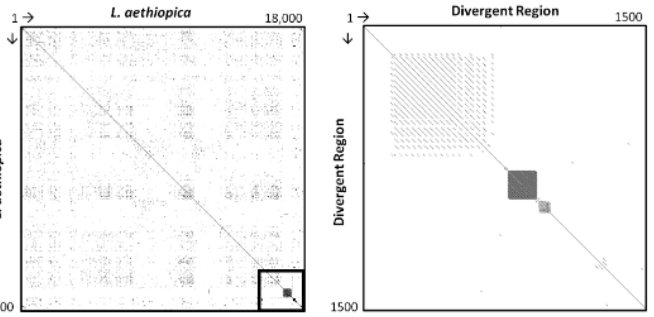
1 →
L. aethiopica
18,000
↓
L. aethiopica
18,000
1 →
Divergent Region
1500
↓
Divergent Region
1500

8

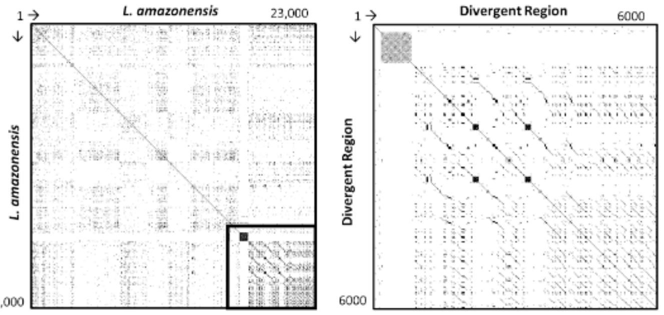
1 →
L. amazonensis
23,000
↓
L. amazonensis
23,000
1 →
Divergent Region
6000
↓
Divergent Region
6000

9

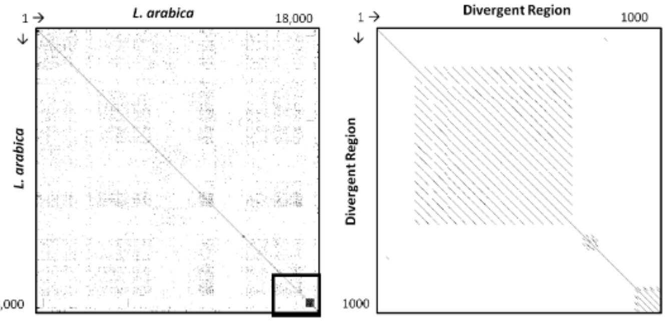
1 →
L. arabica
18,000
↓
L. arabica
18,000
1 →
Divergent Region
1000
↓
Divergent Region
1000

10

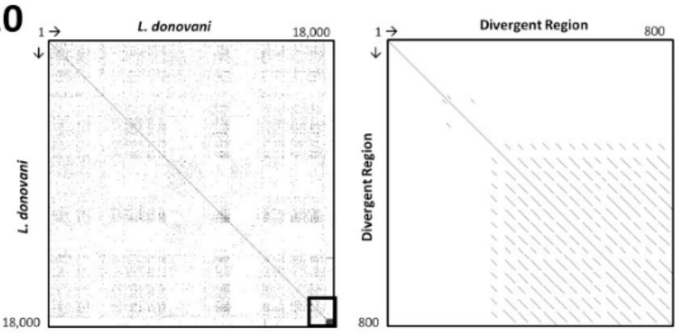
1 →
L. donovani
18,000
↓
L. donovani
18,000
1 →
Divergent Region
800
↓
Divergent Region
800

11

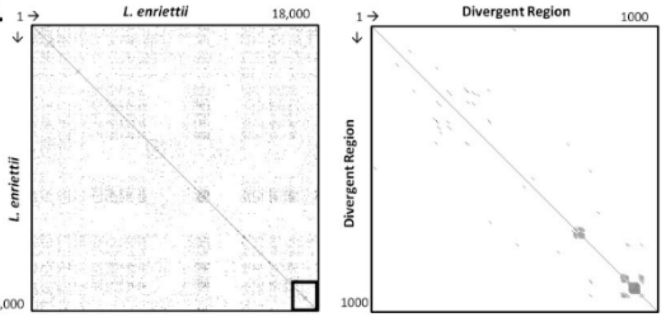
1 →
L. enriettii
18,000
↓
L. enriettii
18,000
1 →
Divergent Region
1000
↓
Divergent Region
1000

12

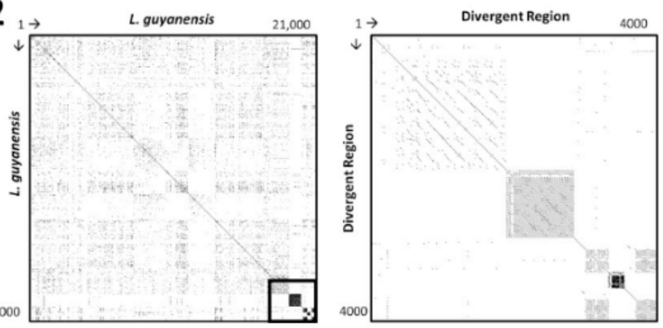
1 →
L. guyanensis
21,000
↓
L. guyanensis
21,000
1 →
Divergent Region
4000
↓
Divergent Region
4000

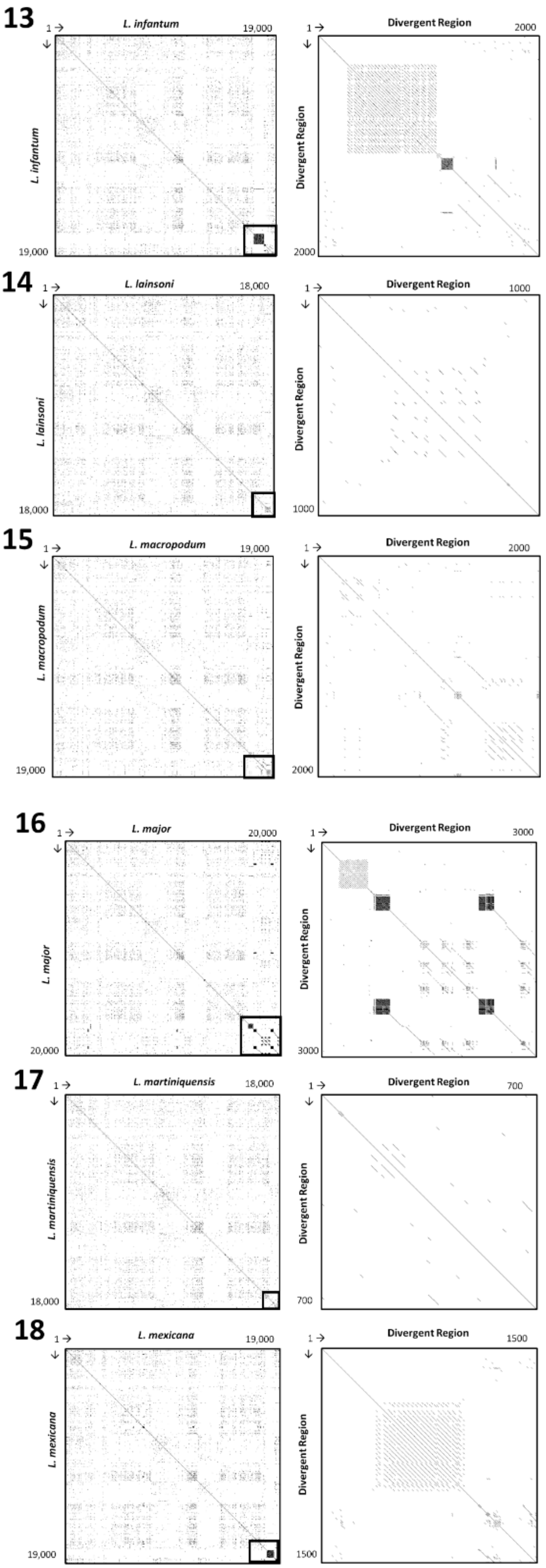

13
1 →
L. infantum
19,000
↓
L. infantum
19,000
1 →
Divergent Region
2000
↓
Divergent Region
2000
14
1 →
L. lainsoni
18,000
↓
L. lainsoni
18,000
1 →
Divergent Region
1000
↓
Divergent Region
1000
15
1 →
L. macropodum
19,000
↓
L. macropodum
19,000
1 →
Divergent Region
2000
↓
Divergent Region
2000
16
1 →
L. major
20,000
↓
L. major
20,000
1 →
Divergent Region
3000
↓
Divergent Region
3000
17
1 →
L. martiniquensis
18,000
↓
L. martiniquensis
18,000
1 →
Divergent Region
700
↓
Divergent Region
700
18
1 →
L. mexicana
19,000
↓
L. mexicana
19,000
1 →
Divergent Region
1500
↓
Divergent Region
1500

19

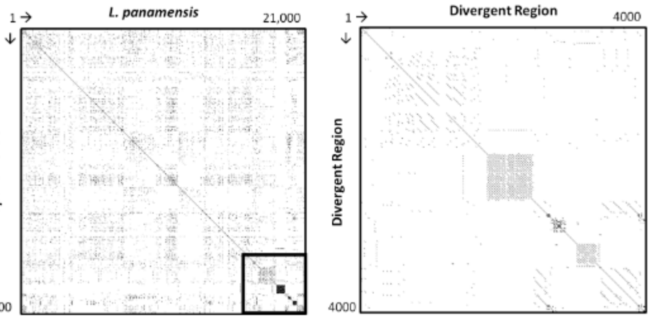

20

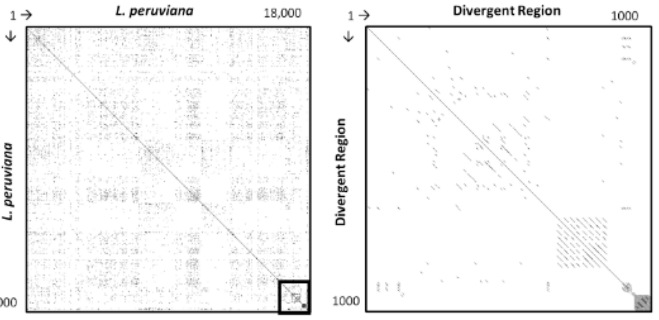

21

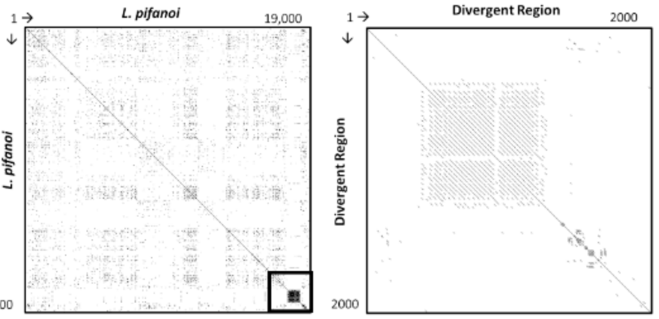

22

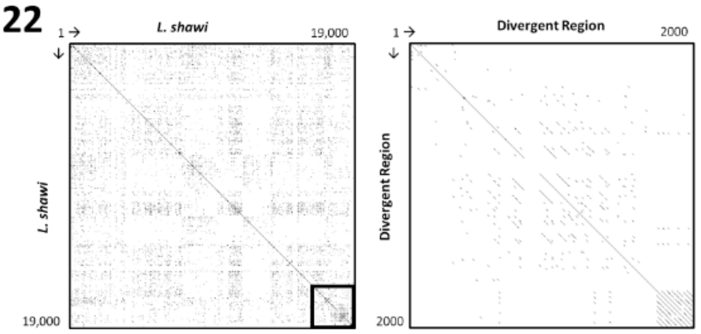

23

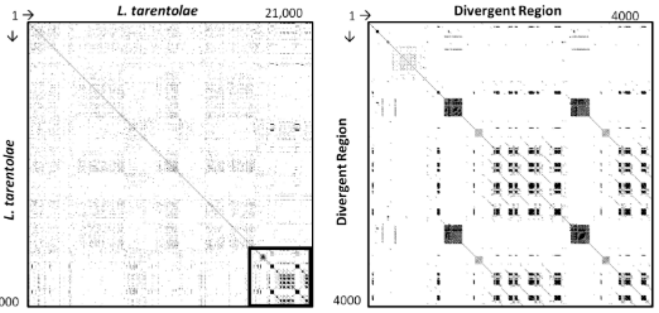

24

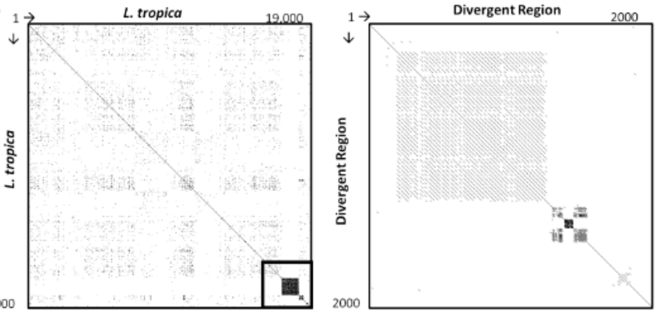

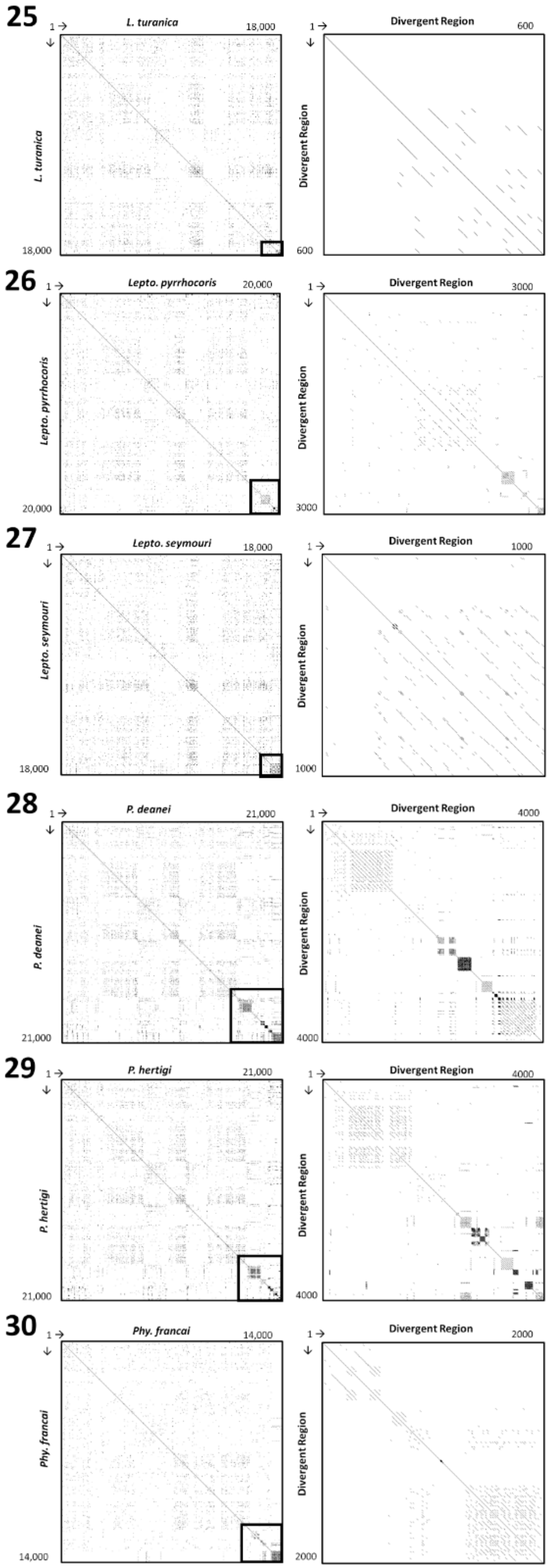

25
L. turanica
1
18,000
18,000
Divergent Region
600
600
26
Lepto. pyrrhocoris
1
20,000
20,000
Divergent Region
3000
3000
27
Lepto. seymouri
1
18,000
18,000
Divergent Region
1000
1000
28
P. deanei
1
21,000
21,000
Divergent Region
4000
4000
29
P. hertigi
1
21,000
21,000
Divergent Region
4000
4000
30
Phy. francai
1
14,000
14,000
Divergent Region
2000
2000

31

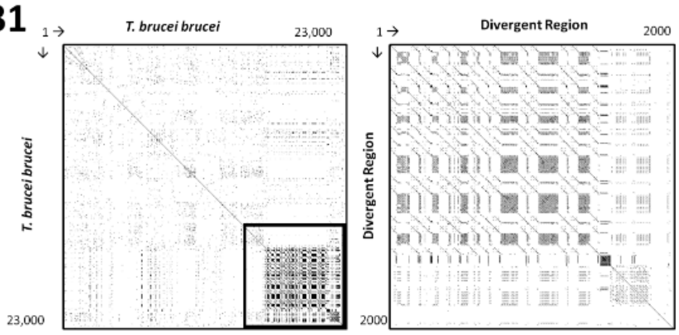

32

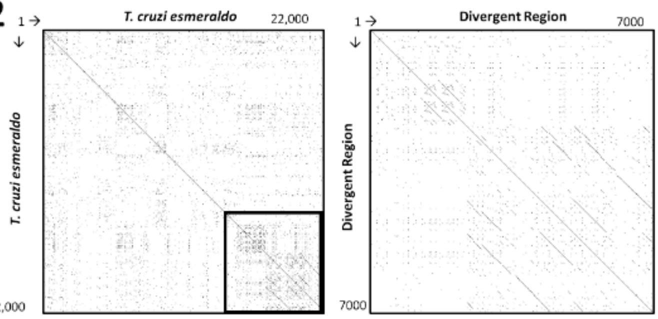

33

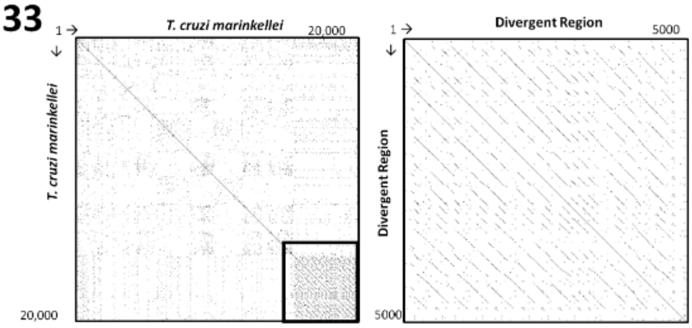

34

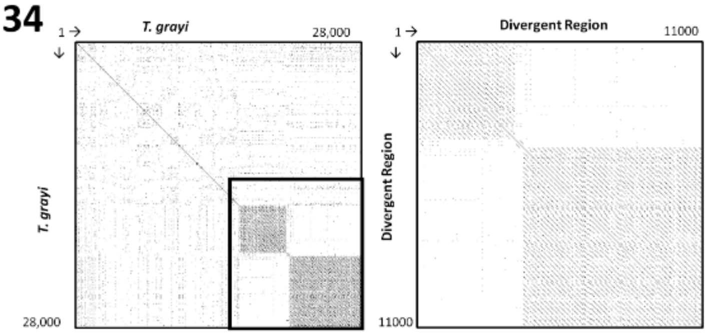

35

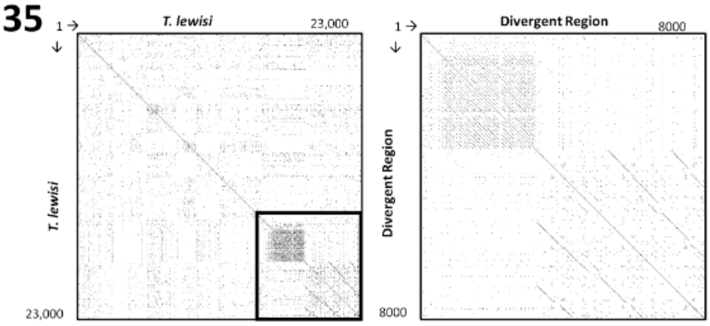

36

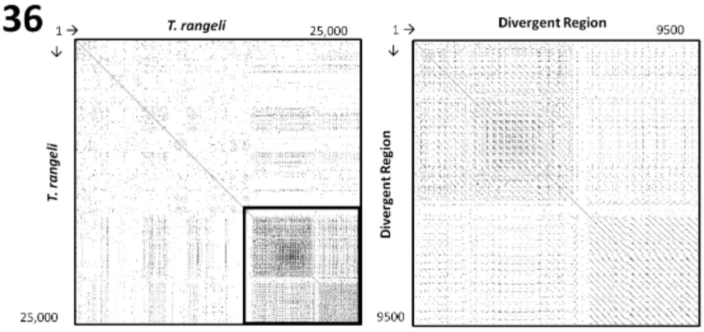

37

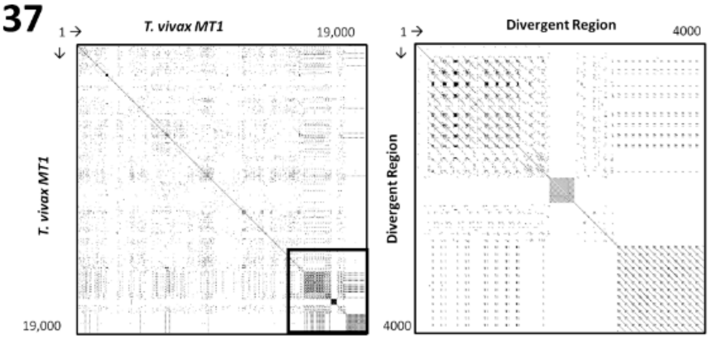

1 →
T. vivax MT1
19,000
↓
T. vivax MT1
19,000
1 →
Divergent Region
4000
↓
Divergent Region
4000

38

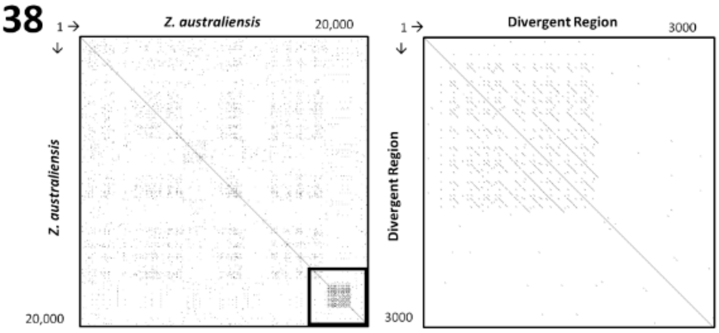

1 →
Z. australiensis
20,000
↓
Z. australiensis
20,000
1 →
Divergent Region
3000
↓
Divergent Region
3000

39

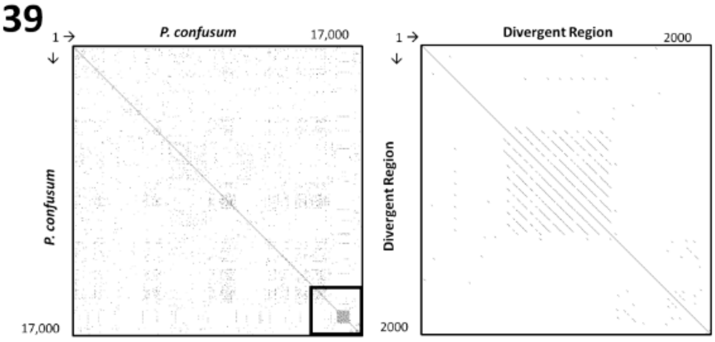

1 →
P. confusum
17,000
↓
P. confusum
17,000
1 →
Divergent Region
2000
↓
Divergent Region
2000

S4 - Analysis of repeated sequences in the maxicircle divergent region

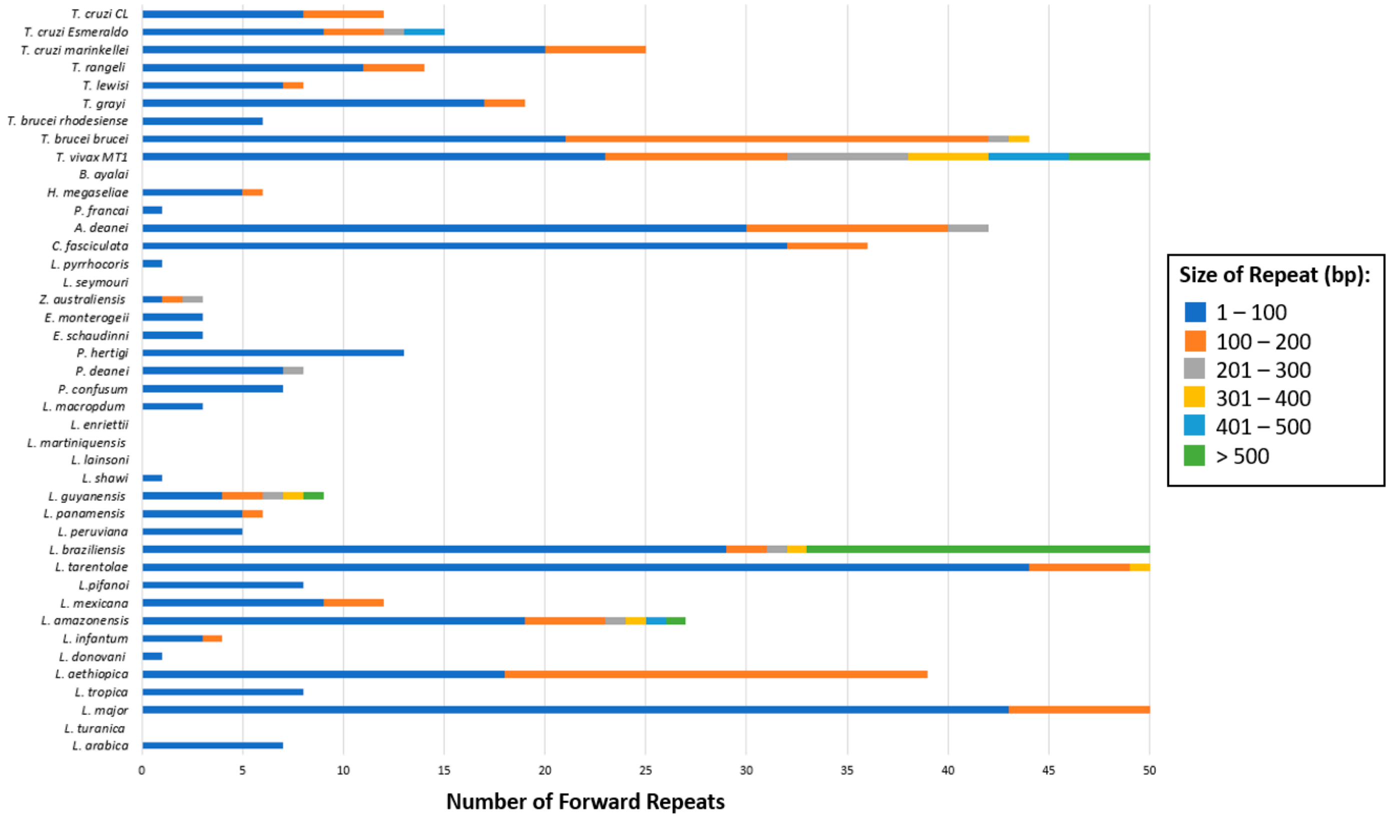

Figure S4-A: Analysis of forward repeats in the maxicircle divergent region 5 trypanosomatid species.

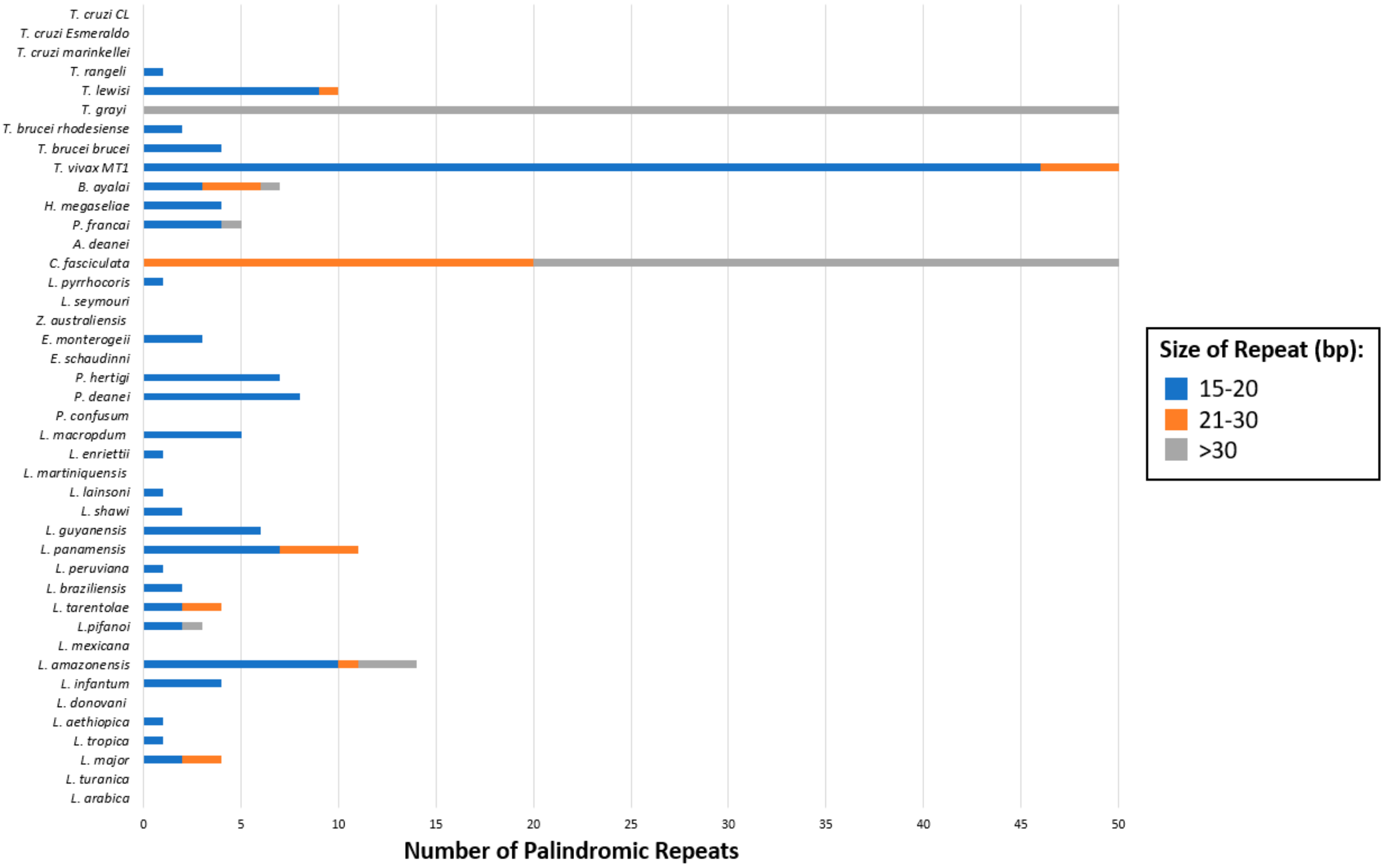

Figure S4-B: Analysis of palindromic repeats in the maxicircle divergent region 5 trypanosomatid species.

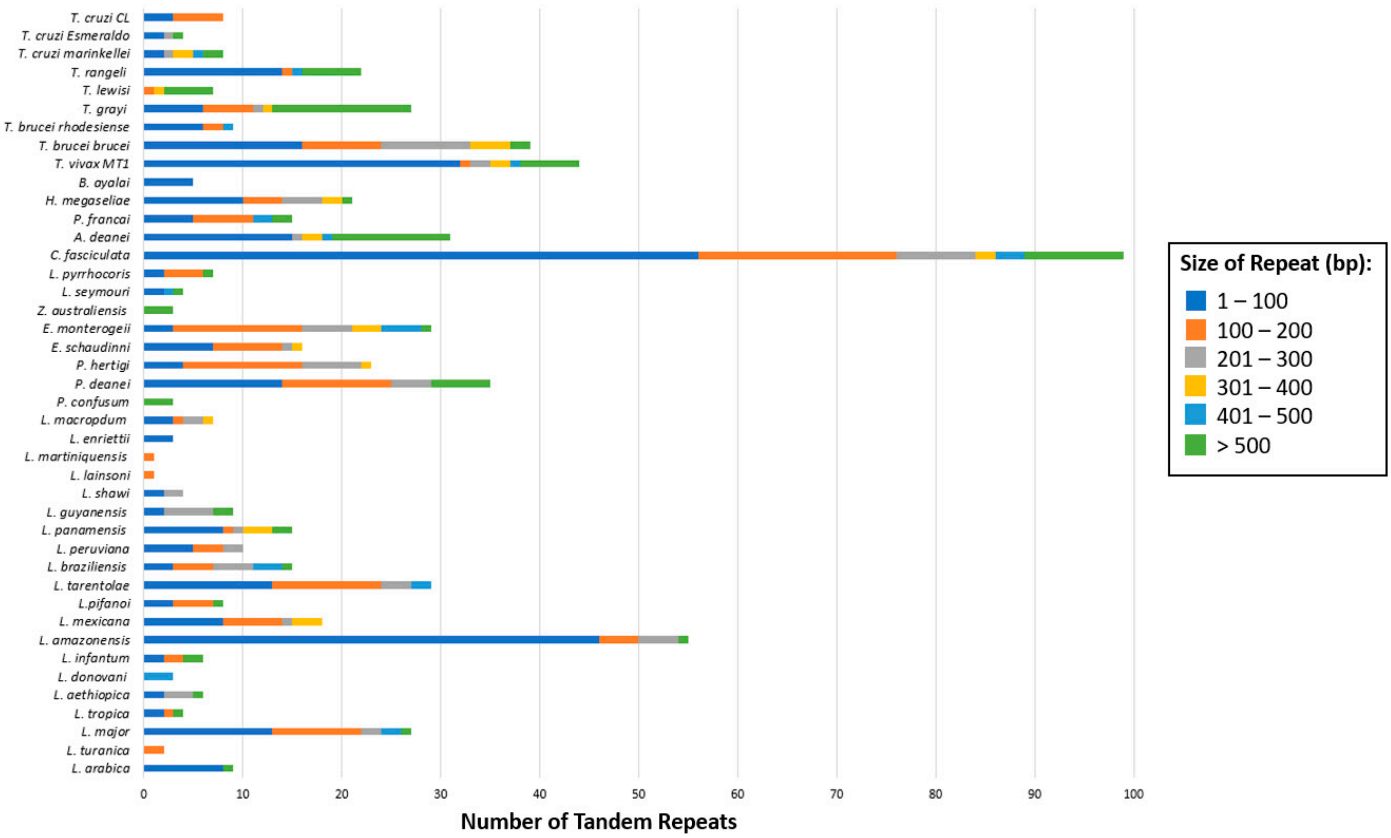

Figure S4-C: Analysis of tandem repeats in the maxicircle divergent region 5 trypanosomatid species.

S5 - Determination of optimal feature/k-mer length (k) for 46 trypanosomatid species.

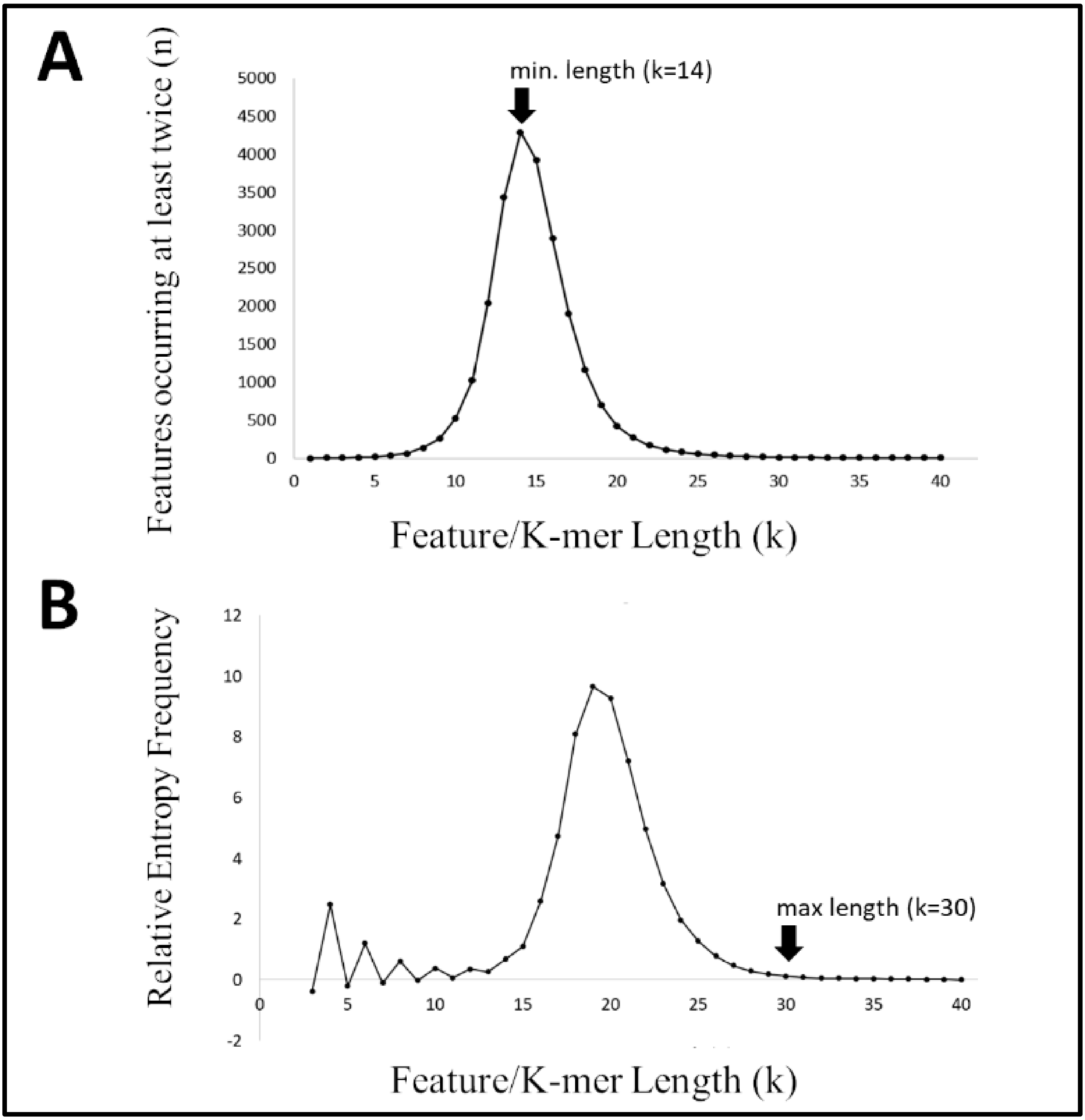

Supplement: Supplementary file 1 [file pathogens-08-00157-s001.zip › Supplementary Files 1 - 5.pdf]
